# Health and Aging: Unifying Concepts, Scores, Biomarkers and Pathways

**DOI:** 10.14336/AD.2018.1030

**Published:** 2019-08-01

**Authors:** Georg Fuellen, Ludger Jansen, Alan A Cohen, Walter Luyten, Manfred Gogol, Andreas Simm, Nadine Saul, Francesca Cirulli, Alessandra Berry, Peter Antal, Rüdiger Köhling, Brecht Wouters, Steffen Möller

**Affiliations:** ^1^Rostock University Medical Center, Institute for Biostatistics and Informatics in Medicine and Aging Research (IBIMA), Rostock, Germany.; ^2^Institute of Philosophy, University of Rostock, Germany.; ^3^Department of Family Medicine, University of Sherbrooke, Sherbrooke, Canada.; ^4^KU Leuven, Department of Pharmaceutical and Pharmacological Sciences, Leuven, Belgium.; ^5^Institute of Gerontology, University Heidelberg, Germany.; ^6^Department of Cardiac Surgery, Medical Faculty, Martin Luther University Halle-Wittenberg, Halle (Saale), Germany.; ^7^Humboldt-University of Berlin, Institute of Biology, Berlin, Germany.; ^8^Center for Behavioral Sciences and Mental Health, Istituto Superiore di Sanità, Italy.; ^9^Budapest University of Technology and Economics, Budapest, Hungary.; ^10^Abiomics Europe Ltd., Hungary.; ^11^Rostock University Medical Center, Institute for Physiology, Rostock, Germany.; ^12^KU Leuven, Department of Biology, Leuven, Belgium

**Keywords:** terminology, health, aging, biological age, wellbeing, biomarker

## Abstract

Despite increasing research efforts, there is a lack of consensus on defining aging or health. To understand the underlying processes, and to foster the development of targeted interventions towards increasing one’s health, there is an urgent need to find a broadly acceptable and useful definition of health, based on a list of (molecular) features; to operationalize features of health so that it can be measured; to identify predictive biomarkers and (molecular) pathways of health; and to suggest interventions, such as nutrition and exercise, targeted at putative causal pathways and processes. Based on a survey of the literature, we propose to define *health* as a state of an individual characterized by the *core features* of physiological, cognitive, physical and reproductive function, and a lack of disease. We further define *aging* as the aggregate of all processes in an individual that reduce its *wellbeing*, that is, its health or survival or both. We define *biomarkers of health* by their attribute of predicting future health better than chronological age. We define healthspan pathways as molecular features of health that relate to each other by belonging to the same molecular pathway. Our conceptual framework may integrate diverse operationalizations of health and guide precision prevention efforts.

## Introduction

For some years, the concepts of *health* and *healthspan* have been advocated as the primary goal of medical diagnosis and intervention [1-4]. Given their importance for national and international allocation of resources in research and care, it is important to define these terms as precisely as possible. In this paper, we suggest a set of operational definitions, including definitions of *health* and related terms such as *wellbeing, biological age*, and *aging*, and we place these into a consistent systematic framework. Our aim in presenting these definitions is to support empirical studies, in particular in health and aging research, and to facilitate the comparability of results. For this reason, we aim for a coherent set of definitions that are practical in the sense that they can be used in actual research contexts. This requires that the definitions can be operationalized, that they are based on a sufficient consensus in the research communities and are sufficiently robust to be applied to different experimental and clinical settings covering molecular as well as higher-level phenotypic phenomena common for a variety of biological species - in particular human and model organisms such as *C. elegans* and mouse.

Specifically, we dissect *health* into a hierarchical system of its various aspects, allowing to analyze its features in detail, and to identify the biomarkers, molecular pathways and corresponding supportive interventions for the various aspects of health. While beyond the scope of the present paper, the inter-related aspects of health that we describe can in principle be scored and weighted, and thus provide a way for the overall measurement and comparison of the health of different individuals. Defining health based on disease and dysfunction, we follow a consensus approach by means of a literature survey. For disease, we employ the World Health Organization (WHO) *International Statistical Classification of Diseases and Related Health Problems*, and for dysfunction, we start with the WHO *International classification of functioning, disability and health*. The latter will then be utilized as background for the review of pertinent papers from health and healthspan research, to systematize our findings. From this consensus, we then derive appropriate definitions of healthspan, healthspan-enhancing processes and biomarkers of health, as well as wellbeing, aging, and biological age. In order to allow the step from prediction to enhancement, we finally distinguish between correlative features on the one hand, and causal features which are potential targets of interventions in order to increase healthspan on the other hand. Our definitions are designed to apply to most animal species, although the literature we surveyed, and thus the operationalization of health we suggest, is specifically targeted to human and the model organisms *C. elegans* and mouse. Overall, we arrive at a framework of definitions, covering states, time periods, associated processes and predictors of future states, as given in Table 1. We suggest that this generic framework of simple and threshold-free definitions of common terms places these into context while still preserving, to a maximum degree, their intuitive meaning.

In this paper, we will first present a framework for the different kinds of terminological categories (states, time periods, processes, predictors). We then define the key term *health* and closely related terms such as *healthspan*. We define the term *survival*, contrast its meaning with health, and propose to integrate both terms under the integrative concept of *wellbeing*. Often used indicators of health such as quality of life, activities of daily living, lack of frailty, or self-reported health (in case of human), and indices such as the *Healthy Aging Index* can then be viewed as projections or surrogates of wellbeing. We further define *aging* as the set of all processes in an individual that reduce its wellbeing, that is, its health or survival or both. Regarding predictors, we define the term *biomarker* (for features of health, survival, or wellbeing) as generically as possible, as a predictor for these features that is better than chronological age. Such a biomarker is a feature itself, and as any feature, it may be composed of more elementary features. We discuss various classes of biomarkers (of aging), considering, for example, causality of various kinds. We define *healthspan pathways* as molecular features of health that relate to each other, specifically by belonging to the same molecular pathway. Precise definitions of other standard concepts such as *biological age* follow naturally.

**Table 1:** Framework of definitions.

	State	Time period	Underlying biological processes	Predictor of future state
Single concepts	**health**	**healthspan**	healthspan-enhancing processes	health biomarkers
	**survival**	**lifespan**	lifespan-enhancing processes	survival biomarkers
Integrative concepts…	**wellbeing**	“wellspan”	wellspan-enhancing processes	**biological age**
… and their opposites	illbeing	“illspan”	**aging** processes	
Baseline reference	baseline organismal state	**chronological time**	average biological processes	**chronological age**

Frequently used terminology that we can fit into our framework is marked in boldface. The terms in the last row, and specifically the term “average biological processes” refer to a specifically selected reference population.

## How to Define Health with Respect to a Reference

Therapeutic interventions affecting aging and health may have different goals. Often, the emphasis of preventive as well as curative interventions was on the extension of *lifespan*. But for most people the mere extension of life is not desirable: if it were possible to live for several hundred years in a vigilant coma, hardly anyone would prefer such a long enduring vegetative state to a normal human life with a much shorter lifespan. For this and other reasons, emphasis has shifted to increasing *healthspan*, i.e., the time period that an individual spends in a state of health. Lifespan is relatively easy to be operationalized. While, from a theoretical perspective, life is both intensionally and extensionally vague at its borders [5], this does not matter much in the context of medical research. For practical purposes, “being alive”, that is, *survival*, can be modelled as a binary state: any individual, as a whole, is either alive or it is not. (We consider only the survival of an individual as a whole, not the life status of body parts like organs, tissues, or single cells). The time period of an individual spent alive is its *lifespan*. Death is the irreversible end of biological life.

In contrast, it is much more difficult to operationalize health and healthspan. For one, the definition of health itself is contested: Is it an intrinsic property of an individual, or is it the extrinsic statistical property of instantiating certain features better than the average of a relevant reference group? Is it a subjective value-judgement, or is it ascribed to individuals in a socio-constructive way [6, 7]? We will argue that an operational definition of health needs to incorporate elements from all these approaches. Second, as one may expect, the features of health turn out to be quite different in humans and model organisms like *C. elegans* or mouse. Third, it is not clear whether there is an irreversible end of healthspan other than death. Often, the healthspan of an individual is assumed to begin with conception or birth and end at some later time. But individuals can have diseases or dysfunctions during their life and then recover; they may even be born with a disease or a dysfunction and then have their health (re-)gained. We thus do not define healthspan as a single coherent time-interval, but allow it to stretch over unconnected intervals, and simply define *healthspan* as the time an individual spends in a state of health, where “health” is, in turn, operationalized as described below. This allows us to stay uncommitted to the question whether it is possible in principle to (re-)gain the state of health.

These problems of the WHO definition motivate an approach in which the severity of any deviation from health is weighted on an individual basis, taking into account that goals may change with time and circumstances. This also holds for the time period in which health is desired. Possibly, some individuals (such as athletes) may want to trade better physical function in the short term for worse health in the long term. Thus, there may be trade-offs between different features of health, as well as between the intensity and the extension of health, given that both cannot necessarily be optimized at the same time. We will introduce the concept of wellbeing (different from the WHO term “well-being” featuring in the WHO definition of health) in order to integrate health (healthspan) and survival (lifespan) according to the subjective weighting of individuals. As our operationalizations of health and wellbeing make use of more features than one, these features have to be weighted in order to be integrated into a single score. Such weighting is necessarily subjective, and different weights may reflect different preferences of different people. In case of non-human animals, weighting is (implicitly or explicitly) carried out by the researcher; here, the subjective view of the researcher replaces the preferences of the individual.

Our definition of health will thus contain a subjective element with respect to certain weighting factors, but the very features that are weighted comprise objectively measurable aspects. Thus, we will not advertise a subjective definition of health [6]. Subjective theories of health define health in subjective terms: Individuals are healthy, according to these theories, if they feel healthy or report to be healthy. Feeling healthy is usually considered a necessary aspect of health, and self-reports are often used to operationalize health. However, subjective aspects cannot be the whole story: Individuals can feel healthy although they have diseases or dysfunctions still unknown to them or ignored by them. In addition, coping strategies and compensation as well as a change in goals and values may influence the subjective assessment of one’s health. Moreover, subjective theories are not feasible for other species like worm or mouse: even if worms or mice had a subjective self-conception of being healthy, they would not be able to self-report their health status at an interview.

In contrast to a subjective approach to defining health, we will define *health* as a state of an individual based on specific objective features, namely* the absence of disease and dysfunction*. As most of these features can be realized in a gradual manner, the question arises where exactly to put the threshold: We need to introduce thresholds in order to distinguish between healthy and unhealthy individuals. In order not to have to introduce arbitrary thresholds, we will refer to the average realization of the features in a certain reference population. Thus, our approach is threshold-free in the sense that we do not set any thresholds a priori; we only provide a recipe for setting these in a generic way.

Furthermore, in view of the controversial discussion found in the literature, we refrain from starting top-down with a new definition of health, for which we then have to find means to operationalize it. Instead, we opt for a bottom-up strategy and first look at how health is *de facto* operationalized in the research literature, and then systematize the findings. A near-consensus in the health literature is that health is a state of an individual that lacks dysfunction and is free from diseases (while it is a matter of debate what exactly counts as a dysfunction or a disease). However, the following issues arise: *Which* functions are these, dependent on species? While the health of humans will in part consist of their capability to exercise higher cognitive functions, these will be irrelevant for worms. *To which extend* does a function need to be exercised, or to which degree does a disease have to lack its manifestation, in order to count an individual as healthy? Finally,* which weight* should be assigned to each of these criteria? As noted, different human individuals will decide on this in different ways.

In order to address these issues, we start with two well-established codified classification systems provided by the WHO. Using the ICD, the International Statistical Classification of Diseases and Related Health Problems, [9], disease is operationalized by criteria to establish that an individual is affected by disease. Using the ICF, the International classification of functioning, disability and health, [10], dysfunction is operationalized by criteria to establish that an individual is affected by dysfunction. While taking the ICD as a given, we filter the definitions of the ICF by their follow-up in the literature on health and healthspan, arriving at a pragmatic community consensus. Starting from the ICF classification, we reviewed pertinent papers from health and healthspan research with respect to how they operationalize health, systematizing our findings according to the ICF classification. In some cases, our review gave us reason to modify the default presented by the ICF classification.

Once the different features have been selected and measured, we can compare the values measured for these with the reference values that are the average in a reference population. For example, we can compare the grip strength of a 60-year old individual with the average grip strength of 60-year olds in the reference population. Depending on whether the value measured is below or above average, optionally considering the variability of values in the reference population, we can assign a score to this feature, and we can consider this individual to be in bad or good health *with respect to this feature*. E.g., a very simple (and often inadequate) scoring would assign -1 to values below average and 0 to values above average.

Using some subjective weighting, we can then integrate the scores for all features into one overall *health score*. This would be done in a standardized way, which reflects the different aspects of health. Such an approach mirrors the use of *qualifiers* in the ICF. The simplest health score would employ equal weighting; if it were based on binary scores, it would amount to just counting the number of diseases or dysfunctions of an individual, based on a list of measured ones. Indeed, dysfunctions are often listed, scored and summed up in the literature, yielding frailty and health scores (as detailed below). In case of disease, the ICD considers disease severity in some cases, but the idea of scoring diseases by severity can be implemented in principle for most if not all diseases. In fact, such severity scores can be based on a calculation of dysfunction due to disease (more precisely, of disability-related sequelae of disease and injury). On this basis, as part of the worldwide GBD (Global Burden of Disease) studies, YLDs (years lived with disability) and HALEs (healthy life expectancies), equal to the sum of the prevalence multiplied by the general public’s assessment of the severity of health loss, were calculated, establishing country-age-sex-year reference population data for sequelae, where same country may or may not imply similar genetics [11].

Given a health score, it is thus straightforward to compare the score of two individuals, or to compare the score of an individual at different times. We can then talk about health in comparative terms, i.e., we can talk about “better” or “worse” health. However, as noted, to define “good” or “bad” health in absolute terms, we need a reference value as a threshold dividing good health from bad health. Full scores of 100% on all features would be required by the WHO definition of health. As this is too strong a requirement, we would like to say that an individual is in good health, if the health score of the individual is above a specific threshold. However, already in the simple case of grip strength a threshold is not straightforward to define in such a way that a grip strength below threshold must necessarily be considered unhealthy. As we strive for definitions that do not require the setting of (arbitrary) thresholds, we refer to a baseline as the reference value also for the health score itself and take note of any deviations. As noted, our standard baseline for defining the health of an individual is the age-matched population average, as it develops along the time axis. Thus, for a reference population, we will consider that its average health develops as a function of chronological time, driven by “average” biological processes (see also Table 1). We take the reference population to be fixed once and invariant thereafter (though we consider various age groups within the reference population); also, we expect that it matches sufficiently well in terms of the years (or, more generally, time period(s)) during which the samplings and measurements are done. (A reference population from the 19^th^ century would not be considered to be a good match for individuals of the 20^th^ century).

An alternative choice is an age-invariant reference population that does not change as the individual gets older, for example, a “young adult” reference population. This choice would allow us to follow an individual on the same scale over time. If the features of this individual stay constant, this may be interpreted positively as “stability”. If an age-matched reference population were used, the change of features then observed for such a “stable” individual would instead be interpreted positively or negatively (depending on how the measurements in the age-matched reference population changed along the time axis, and on how these measurements are interpreted as features of health). For example, if the grip strength of an individual does not change, this observation would indicate “stability”. If grip strength deteriorates in the age-matched reference, however, the relative change in grip strength would indicate an improvement in relative terms [12]. (A related aspect that is beyond the scope of this paper is the need to consider all biomarker measurements on an individual basis, not just with respect to the average in a reference population. One idea here is to employ factors such as genetics/ethnicity or sex to define specific reference populations that are a better match for certain individuals, but their size and therefore the robustness of the average feature measurements based on these subpopulations necessarily becomes smaller, and missing values become more of a problem. For example, to compare two individuals of different regional origin, two different reference populations may be employed, and the resulting relative measurements be compared. Another idea is the consideration of specific composite features consisting of the feature F1 that was measured to estimate health, and, based on some scientific evidence, another feature F2 that is used to elaborate on the difference between the measurement of F1 and the population average given for F1. For example, a genetic feature reflecting low cardiac risk (F2) may suggest that a blood pressure measurement (F1) higher than average does not contribute to a below-average health score for some specific individual, following [12]).

Our threshold-free definition of health is matched, in a natural way, by our definition of a biomarker of health as a predictor for health (see section 4). Quite simply, a *predictor* for health has to predict the future state of health of an individual better than chronological age. This threshold-free definition allows flexibility in the same way as the standard definition of a biomarker of aging with respect to predictions that are better than chronological age [13]. A level of (statistical) significance may be required for the improvement in predictive accuracy, by a more restrictive yet compatible definition. As described, the thresholds for the measurements are by default based on a reference population (see also [14, 15]). Our relative definition of health is compatible with the definition of predictors relative to the baseline of chronological age. Moreover, we do not distinguish linear and progressive aspects of aging; these may be considered in more restrictive definitions (see section 6).

Traditionally, aging researchers were concerned with increasing lifespan; we call the underlying biological processes *lifespan-enhancing processes*. Instrumental for this goal is the search for features that are correlated with the lifespan of an individual, and can thus be used as predictors of survival, that is, as biomarkers of future (residual) lifespan. Such predictors are usually found based on statistical reasoning: What is the statistical life expectancy of an individual with the biomarkers in question? Similarly, the goal of health researchers is to uncover biological processes that enhance health and, thereby, the healthspan of individuals. We call the (molecular) processes resulting in health *healthspan-enhancing processes*. Just as there are predictors of survival (residual lifespan), there are predictors of future health (residual healthspan); naturally, there is a lot of overlap. Furthermore, ideal predictors are to be distinguished from the estimates that may be calculated for these. Along these lines, we suggest that the ideal predictor of both residual lifespan and healthspan may simply be called “biological age” (see Section 4). A similar approach was taken by [16], and the resulting predictor was referred to as “biofunctional age”.

**Table 2:** Features contributing to a definition of health.

Feature	limited to species	pathological	References
physiological function			
*stress resistance*			[17-21], cf.
thermo-tolerance (=heat shock tolerance)			[22, 23]
hypoxic stress tolerance			[24, 25]
osmotic stress tolerance			[26]
oxidative stress tolerance			[19, 23, 27]
metabolic status / *homeostasis*		x	cf.[2], cf. [28 29]
redox status / homeostasis		x	[30, 31]
immune status / homeostasis		x	cf. [20, 32]

physical & cognitive function (=strength and cognition)			
motivated/stimulated locomotion	(worm)		[33]
(motor) balance, dexterity	human/mouse		[34-38]
muscle/neuronal/intestinal integrity		x	[39-41]

physical function (=strength)			
[unmotivated/unstimulated] *locomotion*			cf. [18, 20, 42, 43]
grip *strength*	human/mouse		cf. [20, 44, 45]
*pharyngeal* pumping	worm		[18, 22, 46, 47]
gait speed, chair rising	human/ (mouse)		cf. [20, 48, 49]
muscle integrity		x	[40, 41]

cognitive function (=cognition)			
*sensory perception*			cf. [20, 50-52]
(short-*term) memory*, processing speed	(human/ mouse)		[53-56]
sleep, cardiac rhythm			cf. [20, 57]
executive/verbal function	human/ mouse		[58, 59]
neuronal integrity		x	[60]

reproductive function			
number of offspring			[61-64]
offspring health/survival			[65, 66]

lack of frailty, Healthy Aging Index (and similar), allostatic load; lack of physiological dysregulation, self-reported health, quality of life	(human)		[2, 67-74]

(prodromal) organ/physiological function (heart/cardiovascular, neurological, etc.) (prodromal) paralysis, protein aggregation/plaques	human/animal model	x	cf. [20, 75, 76]

lack of disease and medications	(human)		e.g., [77, 78]

Synonyms are marked by “=”, given in parentheses. Species-specificity noted in parentheses is debatable. Pathological features are features that are predictive of future health problems, but they are not usually regarded as features of health per se.

## Defining, Operationalizing and Measuring Health

Operationalizing health by dissecting it into a hierarchy of its various aspects is a difficult task. However, as described, in the literature on human health the two main aspects of health are dysfunction and disease; both have been codified by standard classifications, the most visible ones being the ICD and the ICF published by the WHO. We wish to do justice to both aspects - absence of disease and dysfunction - by considering both as contributors to health, using an integrated approach.

Based on the ICF and the ICD as a guide, we surveyed the literature and assembled the various ways to operationalize health in both humans and model organisms. The results of this review, presented in Table 2, is an operational consensus definition of *health*, which encompasses the aspects of both disease and dysfunction, and includes integrative concepts such as *quality of life* as well as pathological and prodromal features. Each feature can be operationalized in order to be a useful object of inquiry (see the references in Table 2). Each such operationalization gives rise to a score, possibly a binary one (yes/no). Each feature can be weighted, in order to be integrated with the other features, where the weights may reflect the subjective preferences of the individual or the researcher. The features of Table 2, distilled from our digest of the literature, represent a current, yet limited and biased understanding of health, so they are also subject to change in the light of new scientific findings, and they shall be refined by feedback from the scientific community. Our operational consensus definition of health allows to describe the state of health of an individual, characterized by the features listed in Table 2 and their measurements for that individual.

When defining health by absence of disease and dysfunction, our pragmatic approach to defining disease is based on the adoption of the ICD, using all codes. This may be deemed problematic, because many items in the ICD do not represent diseases. For example, chapter XV (codes O00-O99) concerns “pregnancy, birth and puerperium”, chapter XIX (S00-T98) deals with “injury, poisoning and certain other consequences of external causes”, and chapter XX (V01-Y84) lists “external causes of morbidity and mortality”. For this reason, the ICD is, despite its name, not so much a classification of diseases, but a classification of diagnoses. Nevertheless, such permissiveness is not problematic for us, since we weight the various features of health. While some ICD codes are indeed irrelevant in the light of most non-operational definitions of health, we do not need to exclude these codes beforehand, as this is already accounted for by the fact that these codes are likely to have zero weight in any specific implementation of our approach.

Our pragmatic approach to defining dysfunction consists of adopting the ICF, but only as the first step. Since we are concerned with dysfunction, we only consider the part of the ICF concerning “body functions”, not the other parts on “body structures”, “activities and participation” or “environmental factors”. In fact, the chapter on “activities and participation” is redundant for our purposes, because its entries are mirrored in the chapter on “body functions” except for some specific human-related aspects (see also [79]). With the ICF list of body functions in mind, we surveyed the literature, and collected the hierarchical framework of features of Table 2 that can be mapped to the ICF codes in a consistent fashion.

Specifically, in Table 2, the notion of physiological function as found in the literature includes many ICF body functions, such as *functions of the cardiovascular, hematological, immunological and respiratory systems* (ICF, Body functions, chapter 4),* functions of the digestive, metabolic and endocrine systems* (ICF, Body functions, chapter 5), *genitourinary function* (ICF, Body functions, part of chapter 6), and* functions of the skin and related structures* (ICF, Body functions, chapter 8). The notions of physical and cognitive function as found in the literature include* neuromusculoskeletal and movement-related functions* (ICF, Body functions, chapter 7) and, more specific to cognitive function,* mental functions, sensory functions and pain* (ICF, Body functions, chapter*s* 1+2) as well as* voice and speech functions* (ICF, Body functions, chapter 3*).* Finally, the notion of reproduction from the literature includes, naturally, *reproductive functions* (ICF, Body functions, part of chapter 6). Notably, the ICF does not list any important body functions that we miss in our framework, which indicats that our list of features is likely to be complete for our purposes.

In summary, our literature survey of health and healthspan shows that health is operationalized in terms of stress resistance and homeostasis (which we summarize as physiological function), strength (physical function), cognition (cognitive function), and reproduction, as well as in disease-related and integrative terms, see Table 2. Reassuringly, this set of higher-level terms matches closely the NIH toolbox approach that distinguishes four major domains of function: cognition, motor, sensation, and emotion [80]. It also resembles closely the five domains constituting *intrinsic capacity*: locomotion, sensation, cognition, psychological issues and vitality, where vitality unfolds into hormonal and cardio-respiratory function and energy metabolism [79]. Similar to our approach, the latter approach is also making use of the ICF, with a focus on body functions, and it can be extended by extrinsic factors, defining the more general term of “functional ability”.

Recently, the combination of *strength and cognition* (physical and cognitive function) displayed in Table 2 gained popularity. In *C. elegans* healthspan research, health is now often operationalized in the form of “stimulated locomotion”, which can be clearly distinguished from locomotion that is just due to the search for food [43]. In human, “cognitive frailty” was proposed to cover both aspects [81]. The corresponding term *strength and cognition* refers to both, *strength* and* cognition*, giving rise to a hierarchy reflected by the table. The other hierarchy-generating properties in Table 2 are the various specializations of physiological, physical, cognitive and reproductive function. We also included histological and molecular features that are called “pathological” and that are predictive of future health problems, although they are not usually regarded as features of (worse) health per se. For example, no individual suffers directly from microscopic lesions in muscle tissue, or from elevated values of cholesterol or prostate-specific antigen, or from some specific variant of the APOE or BRCA gene; instead, “healthy” measurements of these features are biomarkers of health (cf. Section 4). Along the same lines, we consider pathological features that are early indicators of the onset of specific diseases (“prodromal” features, e.g., protein plaques indicative of Alzheimer’s disease).

Table 2 includes dysfunctions, as well as various integrative approaches towards listing and indexing them, such as (lack of) frailty, “healthy aging” indices and the like. Such indices often include features on various levels of abstraction, but a rigorous justification for a specific selection of features is usually lacking. For example, frailty is defined as a state of reduced physiological fitness that includes multimorbidity, functional limitation, and geriatric syndromes, representing a compendium of interacting factors contributing to poorer health outcomes [80]. There are two more widespread definitions of frailty by [67, 68], but there is still a lack of consensus [82]. Further indices were introduced with an emphasis on “healthy” or “successful” aging, for example, the *Healthy Aging Index* by [69], the *Successful Aging Index* by [70], or the *Healthy Aging Score* by [71]. These indices include features from the sociodemographic domain partly based on self-assessment, disease-related scores such as disease counts, some laboratory markers such as blood pressure, and some examination scores such as the *Mini-Mental State Examination* test result. As another example of an integrative concept, *allostatic load* is based on laboratory markers [72]. A lot more indexes were developed, recently reviewed by [83], most recently encompassing multiple blood-based biomarkers [84], clinical and blood-based biomarkers [85, 86], functional measures and questionnaires [87], multimorbidity [88], or combinations of these [89].

Although the ICD and the ICF are intended to be complementary [90], some overlaps between features of disease and dysfunction may be identified by careful inspection. (For example, the German modification of the ICD comprises several codes for dysfunctions (“Funktionseinschränkungen”, functional limitations), in its chapter XII, codes U50-U52. These codes are not contained in the international WHO version of the ICD, and they are intended to be applied for the initial period of inpatient treatment only). Thus, there is threat of double counting dysfunctions by having codes for them not only in the ICF, but also in ICD. This can be avoided by identifying the respective terms and mapping them to each other. The same holds true for overlaps among single features and integrative ones, and for overlaps among the latter.

In the last row of Table 2, we consider disease and medication. As described, we define diseases pragmatically as anything that is codified by the ICD. Intake of medications is, of course, neither necessary nor sufficient for having a disease. It can, however, be used as a proxy for information about health. Moreover, it is possible to map the feature-based descriptions available for many diseases to animal species if these can also be identified and scored in these species [91]. Similarly, the feature-based descriptions available for human dysfunctions can often be mapped to similar or even identical descriptions of animal dysfunctions.

While health and survival may be contrasted, these two concepts may also be integrated by taking a weighted average, as motivated by [92]. We suggest that the weighted average of an individual’s healthspan and lifespan is the best objective measurement of success of any healthcare intervention. Naturally, the weighting factor for health on the one hand and survival on the other hand will be subjective (as described in Section 1). Our short term for the *weighted average of health and survival* is *wellbeing*, which refers to health only if the weight for survival were zero, and vice versa. For *wellbeing* as the state, we propose to name the associated time period the “wellspan”, and the underlying processes “wellspan-enhancing processes” (see Table 1). The processes that are reverse to “wellspan-enhancing processes”, that is, the biological processes that reduce wellbeing, have a standard term, which is *aging*. In other words, we propose that *aging* (which is a process) is simply the aggregate of all processes that reduce future wellbeing. The definition of aging is as contested as the definition of health (see, for example, [93, 94]). We think that our definition, as the aggregate of the processes that reduce health and survival, matches the intuitive meaning of the concept. We also claim that the concept of wellbeing matches closely the intuitive meaning of the WHO definition, and it covers any changes positive or negative for health and survival that are happening to an individual, including, e.g., the acquisition of “wisdom”.

The state that is the opposite of *wellbeing*, and that is caused by aging, may be called “illbeing”; the corresponding time period is the “illspan” (see Table 1). The sum of the wellspan and the illspan of an individual is its lifespan, and any predictor of illbeing as well as of wellbeing must predict the same entity. As we will see in the next section, the best possible integrative *estimate* predicting the future health and survival of an individual is *biological age.* In the literature,* biological age* also refers to any *estimate* of *biological age*, and not just to the idealized concept of its best, or perfect, estimate.

## Predicting Health

The prediction of health, survival or wellbeing is often based on chronological age alone. Such a prediction is often a good one, but it is not the best one possible, as it cannot account for differences among individuals of the same age. Information about the actual state of the individual can make the prediction more precise. In our generic, simple and threshold-free framework, a *biomarker* is a feature of an individual that allows prediction of another feature of the same individual. This definition avoids hard-to-define terms such as “indicator”, which normally feature in definitions of “biomarker”, e.g., in the NIH definition (“a characteristic that is objectively measured and evaluated as an indicator of normal biological processes, pathogenic processes, or pharmacologic responses to a therapeutic intervention”) [95], or the definition by Merriam-Webster (“a distinctive biological or biologically derived indicator (such as a metabolite) of a process, event, or condition (such as aging, disease, or oil formation)”). Furthermore, adapting the approach of [13], a *biomarker of health* is any feature of an individual that predicts a temporally later feature of *health* better than chronological age, and a* biomarker of aging* is any feature of an individual that predicts a temporally later feature of illbeing better than chronological age. These definitions are not cyclic. Since wellbeing and illbeing are opposites on one and the same dimension, a *biomarker of aging* predicts the corresponding features of *wellbeing* equally well.

Further, considering that *biological age* is supposed to predict the future wellbeing of an individual (referring to the weighted average of health and survival) in the best possible way, it is a biomarker of aging, and it is the best composite biomarker imaginable if it could be estimated without error. Biological age is a concept found frequently in the literature (see, for example, [96, 97]), though it is often not explicitly and precisely defined. The closest approach to ours that we could find is provided by [16], where the authors define “biofunctional age”, in a similar fashion. Our definition thus fills a void, while preserving the intuitive meaning of the term. In our framework, aging increases the biological age of an individual, and biological age predicts wellspan (healthspan and lifespan) best. This is because we analyze aging as the aggregate of all processes reducing an individual’s wellbeing, and whatever changes a feature of wellbeing, also changes the ideal predictor for this feature. As biological age is the ideal predictor for wellbeing, aging must change biological age.

In practice, the biological age of an individual is represented by a specific numerical value that is estimated based on some features of the individual. It is often estimated in years - with the idea that in a baseline population of individuals, individuals with a similar biological age have a similar expected (residual) wellspan. To estimate it in the best possible way, all features of the individual that are contributing independently would need to be considered. Of cause, chronological age is an important contributor to this estimation, even though, by definition, it cannot be a biomarker of aging; a composite biomarker of aging such as biological age can therefore include a significant component that is not a biomarker of aging. And indeed, predicting a feature better than baseline best starts with that baseline, improving upon it.

In general, biomarkers are identified based on cross-sectional or (preferably) longitudinal cohort data, where features of individuals are measured over time. Whenever there is a time gap between measurements, the biomarker attribute (of predicting the future better than chronological age) may be tested. For any individual (which does not have to be a member of the cohort), the biomarkers we are interested in predict its wellbeing (health and survival) better than chronological age. Biomarker measurements that are predictive for some feature in a population do not have to be necessary, nor sufficient, for that same feature for a particular individual. For example, taking for granted that high blood pressure is a good biomarker for shorter lifespan and for cardiovascular disease, it is possible that a particular individual has high blood pressure but still enjoys a long lifespan without cardiovascular disease (because of other factors with protective influence), and another particular individual may feature short lifespan and cardiovascular disease without having high blood pressure (because of other factors with negative influence). However, a high or low biological age is based on the result of the measurement of the widest possible variety of molecules and functions, so that its prediction of wellbeing cannot be overridden.

Features, and biomarkers in particular, can further be classified on the basis of the following questions:

1. *Is it an intrinsic feature?* Features may be *intrinsic* or *extrinsic* to the individual for which their value is measured. Intrinsic features include genetic and epigenetic ones; for humans, these also include behavior and lifestyle decisions. Extrinsic features include environmental (and social) ones, as well as prenatal ones. Both types of features are profoundly interconnected [79]. Given these interconnections, we designed our set of definitions to be valid for intrinsic and extrinsic features, even though their relevance is much higher for intrinsic features, see also the Discussion
2. *Is the feature time-invariant or role-switching?* Features can be classified according to the periods in the life of the individual in which they are predictive. Thus, they may be biomarkers across the time axis of the entire life of an individual, or they may be predictive only during selected time periods. In fact, biomarkers may be time-dependent, up to the point that they may be “role-switching”, that is, predictions of health or survival based on a high biomarker measurement may first be negative, but then turn positive, or vice versa, as an individual gets older [98]. Generally, our definitions are supposed to be valid at young age, though their relevance is higher at middle and old age
3. *Is the feature predictive for itself?* Biomarkers are usually reflexive, so that the current measurement of a biomarker predicts its own measurement in the future
4. *Is the feature diagnostic or theranostic?* Features can be classified according to their role as “prognostic” or “predictive” tools. Diagnostic (also known as “prognostic”) biomarkers can help to set up a diagnosis, that is, they are simply predictors of future health or survival. Theranostic (also known as “predictive”) biomarkers can be used as a guide to find an appropriate therapy or intervention as well
5. *Does the feature have a causal influence?* A causal relationship is necessary between a biomarker and the features of health, survival or wellbeing that it predicts, if our aim is the identification (but not necessarily the monitoring) of interventions. However, prediction “better than chronological age” does not necessarily imply causality, not even partial causality (that is, being one cause of many), with respect to the processes of aging. A biomarker may thus be purely correlative, but by Reichenbach’s common-cause-principle [99] it then should be the downstream consequence of another feature that is (partially) causal; otherwise it could not be a biomarker. A standard example for a pure correlation with age is the possession of grey hair, which is not supposed to cause aging processes in itself, even though, strictly speaking, it may do so by causing a depressed state or other psychological feedback effects that may be causal to aging. Guided by utility, we are most interested in features that are at least partially causal for aging, that is, features that are part of the causal *basis of aging*; popular examples are the so-called hallmarks of aging [100], or what became known as inflammaging [101]. There are a few examples of features related to age that are not predictive for any feature of wellbeing. These features are not biomarkers of aging, being not even partially causal, and not downstream of something causal for any feature of wellbeing. The racemization of amino acids in teeth may be cited as one of them [102]. Such racemization is due to the progression of chronological time, and it has no causal consequences. It is, thus, only a biomarker of *chronological* age. The accumulation of DNA mutations, however, must be considered to be a biomarker of aging, even if the underlying processes were purely chronological, because they have deleterious consequences. In general, we can expect strong correlations between wellbeing, health and survival, but any causal links will be complex, see also [103]
6. *Is the feature easy to measure in practice?* A feature should be easily measurable repeatedly, and the measurement should not influence health or survival by itself, and it should yield comparable results in human and other animal species [13]

## Enhancing Health

As noted, any predictive feature of an individual can serve as a biomarker, which may be molecular (genetic variation, genomic methylation, gene/protein/metabolite abundance, *etc.*) or high-level phenotypic (blood count data, blood pressure, grip strength, anthropometry, *etc.*). Similarly, the healthspan-enhancing and lifespan-enhancing processes as defined above, just as their reverse, that is, aging, are associated with most aspects of its biology, so that it is impossible to strive for a comprehensive description. Nevertheless, we can define the *causal molecular basis of aging* as all features of aging that are both causal and molecular. In fact, both aspects of wellbeing, health and survival, have a molecular basis, and the processes leading to these states have such a molecular basis as well, and so does wellbeing. In case of processes, their molecular basis can consist of composite differential features that are measured as changes in the measurements of features [104]. Like all features, also differential ones may be biomarkers as defined above. Only biomarkers that are part of the *causal molecular basis of aging* can be molecular targets of intervention. Healthspan-enhancing or lifespan-enhancing processes entail maintenance, repair, rejuvenation, as well as the reversal of specific types of hypertrophy or damage, of unreliability and deterioration [102, 105-107] that, as a consequence, move the state of the individual closer to complete health, as defined here, and as defined by the WHO, or that change the state of the individual so that death is occurring later. Naturally, there is a lot of overlap between healthspan-enhancing and lifespan-enhancing processes.

Healthspan and lifespan are often contrasted, and the causal processes resulting in health and survival overlap only partially ([20, 108] and references therein). For example, an individual may suffer from a serious neurodegenerative disease, but survive for a long time. Such a state of disease often consists of a long time spent in (subjectively) worse health. Thus, worse health does not necessarily imply shorter survival, and, in terms of time spent by the individual, “healthspan” and “lifespan” may differ. Naturally, at any time an individual is healthy, it must be alive. Hence, the healthspan of an individual is necessarily included in its lifespan. However, this inclusion relation is not preserved on the level of causal influences. While survival is influenced by affecting health, positive or negative influences on health may not influence survival with the same strength, or may not influence survival in the same direction, and vice versa. Thus, “causal feature for health and survival” is not synonymous with “causal feature for health”: Processes and interventions that positively influence lifespan may be detrimental to healthspan, and vice versa. For example, processes of antagonistic pleiotropy [109] improve health in early years, and reduce survival (and health) in later years. Aortic aneurysm has a much stronger influence on survival than on health, as it is often asymptomatic. On the other hand, dementia usually has a much stronger influence on health than on survival. In turn, treatment of aortic aneurysm by surgery may improve survival at the expense of health, and treatment of dementia may be indicated even if it causes a significant reduction of survival, but an improvement of health.

Finally, given relationships between features, such as molecular interactions, we can assemble sets of related features into (molecular) *pathways.* Although the boundaries between such pathways are ultimately arbitrary, the identification of pathways, and of the interaction (crosstalk) between these, has become a common concept. In our case, some pathways can be labeled as wellspan-enhancing, healthspan-enhancing [110], or lifespan-enhancing pathways, depending on whether the features making up the pathway are related to wellbeing, health or survival. In general, aiming to be threshold-free, a “health relatedness score” can be assigned to every pathway. Nevertheless, in practice, we may still label some pathways as being, e.g., healthspan-enhancing, and others as not being healthspan-enhancing. This labeling may be done based on a threshold on the predictiveness of the features making up the pathway. In turn, we may start with features of health, and construct pathways starting with these. The relationships between the (molecular) features may consist of sets of molecular interactions (for example, protein interaction or gene regulation, documented, e.g., in KEGG pathways [111] or other correlative or causal dependencies). As molecular pathways may interact themselves, their interactions can be described by pathway maps, yielding healthspan pathway maps. The net result of their interaction determines the progression, slowdown or reversal of wellbeing. Thus, [112] started with sets of molecular features (that is, genes that are likely involved in health in a causal fashion), and healthspan pathway maps were constructed for human (and *C. elegans*), based on molecular interaction data. A small number of interacting genes was added to the starting sets, so that at least the majority of genes can be assumed to be causal for health, and the pathways as well as the map between these were then based on a clustering algorithm applied to the molecular interaction network already known for the genes based on other public data.

Relationships between features, and specifically between biomarkers, may consist of relationships among higher-level phenotypic features as well as molecular ones at the same time, based on measuring their correlation [113]. Often, molecular biomarkers are used to predict higher-level phenotypic features. However, a higher-level phenotypic biomarker may also predict a molecular feature better than chronological age. Then again, for practical reasons, we define health by features of relevance to the individual, and these are usually phenotypic, and we strive to find biomarkers as predictors that are easy to measure and yet provide prediction potential for the future state of the individual, and these are often molecular.

## Discussion

In this paper, we describe how health and healthspan can be operationalized for health and aging research. Based on a literature review, we provide a framework for generic, simple and threshold-free definitions of health and health-related terminology. Our definition of health comprises various elements that are often dispersed over distinct approaches to defining health, namely, (1) objective features like the lack of dysfunction and disease, (2) subjective weightings, and (3) the reference to the statistical average in a population. This way, we are able to integrate various operationalizations from the literature into a joint framework. We are optimistic that future operationalizations can be aligned to our framework, thus extending its scope and semantic expressivity. In particular, we expect to incorporate further feedback from the various research communities. We hope that such community feedback can also help to minimize our investigator bias.

We intend our framework as a means of integration for previous, present and future operationalizations of health. While we are striving for a framework of definitions that are as generic, simple and threshold-free as possible, we allow to design more restrictive frameworks by placing constraints on some of the definitions. In some cases, the more restrictive instantiations of our framework are more intuitive, but also less simple. In particular, we consider intrinsic as well as extrinsic features of health, while a restriction to intrinsic features that are contained within the individual may be more intuitive. Also, in our generic, simple and threshold-free framework, a biomarker is just an (intrinsic or extrinsic) feature of an individual that predicts another (intrinsic or extrinsic) feature of the same individual at a later time-point.

Our list of health features (Table 2) is long and complex, and it seems to be too unwieldy to be handled. We do, however, not want to imply that future studies of health or healthspan need to take into account all features in the list at the same time. To the contrary: As the features will be combined with a subjective weighting, those features that are not made use of in a given study can simply be combined with a zero weight in order to neutralize them. Indeed, a specific set of features can be selected, scored and weighted to yield a single score describing a specific aspect of health. Single scores can then be combined, considering various specific aspects of health, and eventually covering all features of Table 2. The more these specific sets of features and the subsequent scoring follow a standard based on a consensus among researchers, the higher the comparability of results from different experimental studies.

Admittedly, it is a difficult business to reduce the complex state of an individual to one single number, and it goes without saying that this comes at a price. First and foremost, a lot of information is lost on the way. For example, we do not capture the state of single organs or organ systems. An individual may have a biologically young skin, but a biologically old heart. But this is not problematic, as the point of the procedure is to distill the biological age of an individual, i.e., the best possible predictor for health and survival - and in this respect, the biologically old heart will probably weight more than the biologically young skin. While we try to avoid arbitrary thresholds, we do need to work with subjective weightings and reference populations, both of which allow for many variations. We see this as a benefit of our approach, as variation in both weighting and reference population may give rise to different kinds of analyses. The default choice of the reference population for health feature measurements is an age-matched population, i.e., we compare the data of an individual with a reference group whose members have similar chronological age as the individual under study. But it might also be very useful to compare results with a reference population that is matching closer the genetics of the individual under study. Still another option is to compare the results from individuals of any age with a reference population of a fixed standard age, e.g., a population of young adults (see Section 2). In any case, the choice of the reference population is an issue that shall be explored further.

Our definition of aging is very broad. According to our definition, any biological process that reduces health or survival will count as an aging process. We think that diverse processes such as the disease course of progeroid syndromes, preterm birth, the development from puberty to adulthood, traffic accidents, moving to a war zone, or losing one’s social interactions are all aging processes. This implies that we operate on a very broad definition of “biological” here, but we are convinced that all of these processes have at least a biological component. Specifically, there is no doubt that the disease course of progeroid syndromes such at Hutchinson-Gilford progeria consists of aging processes, reducing health and survival. Preterm birth and its consequences is actually quite alike progeroid syndromes, in that they include aging-related processes in basically the same way. In both cases, health and survival tend to be reduced, and the underlying molecular biology even features common molecular processes, e.g., in case of mandibuloacral dysplasia [114, 115] and Marfan lipodystrophy syndrome [116].

Taking the broad view, development from puberty (at which time human mortality is at its lowest in many countries) to adulthood also features some aspects of aging, that is, reductions of health or survival, e.g., due to risk-taking behavior that has at least in part a molecular or genetic determinant. In late adulthood, the relevance of risk-taking usually diminishes, but at the same time the effectivity of the response, in terms of cognitive abilities, goes down. By the same argument, almost all kinds of accidents, war- or crime-related death have biological components, even if non-biological external causes (like a brake malfunction) are more salient. Along the same lines, we can include social processes within our definition of aging processes - although social processes are extrinsic to the individual, and their effects on the individual are mediated by internal psychological processes in a fashion that may be specific for the individual, they are biological in the broad sense that they involve, in one way or other, genes, brains, and hormones. In the generic framework proposed here, the absence of social isolation, poverty, *etc.*, are thus features of health, in line with the notion of *functional ability* [79] and the WHO “World Report on Ageing and Health” [117].

In fact, subjective aspects arise specifically for any definition of concepts relating to human. For example, aspects of social life are particularly prevalent features of human health, and we consider these as well as some of their cognitive prerequisites in Table 2, specifically as part of some of the integrative features. In particular, social contexts can turn the presence of a disease, which primarily has a negative effect, secondarily into an advantage, that is, into a *secondary disease gain.* For example, a certain disease may exempt from military service and thus indirectly prolong the life of the diseased, or it may lead to more attention by relatives and friends. Human beings are able as well as forced to integrate various (even pathological) circumstances into a dynamic system of judgements, decisions, values and goals. Considering an individual with a chronic condition, e.g., chronic heart failure, a disease which will progress over time, the goal of a long lifetime may be a function of a composite of - maybe contrary - wishes, beliefs, values, and goals that must be rationally and emotionally integrated into the current and future life course. Thus, quality of life is usually influenced by personality, life experience, cultural factors, personal (including financial) resources, social support networks and other unique life circumstances [118].

As we explained above, our definitions do not exclude that external accidents as well as war or crime-related misfortunes are aging processes, if they result in disease, dysfunction or death. Our main rationale for this broad view is to avoid arbitrary thresholds; in our view, *all* processes reducing health or survival should qualify as aging. To a certain extent, traffic accidents and war damages are externally caused, but they are not totally independent from intrinsic, biological features. For example, traffic accidents are more likely given risk-seeking behavior, and they are less likely given good cognitive abilities, both of which in turn may well have genetic roots and vary with age. Nevertheless, our framework is flexible to accommodate a restricted set of definitions, where all features considered must be intrinsic to the individual, and all extrinsic features are excluded. It would then fall to the proposer of such a restricted framework to delineate between these two classes of features.

We defined aging as those processes that contribute to disease, dysfunction, or death. As these processes start early in life and accumulate over time, some authors propose to define aging as a disease [119, 120]. The ICD-11, the new release of the ICD, will contain an extension code “Ageing-Related” (XT9T) for ageing-related diseases. This in itself does not prejudicate whether aging is a disease or not: the diagnosis that some disease is aging-related is not tantamount to the claim that aging itself is a disease. We defined aging as the aggregate of all processes in an individual that reduce its wellbeing. As wellbeing aggregates health and survival, and health is defined as a lack of disease and dysfunction, this implies that aging is a cause of disease *or* dysfunction *or* death. Given our definition, it is thus much more natural to conceive of aging processes (as defined by us) as causes of disease, rather than being diseases themselves [121]. However, there would not be any logical inconsistency if aging were a disease, for among the causes of disease, dysfunction or death may well be diseases, while not every cause of these three need to be disease.

In our present analysis, we ignored most of the work on the demography of aging. For example, some demographers distinguish “true” progressive aging from linear processes related to wear and tear, or to disease. Some demographers thus investigate mortality patterns using Gompertz’ law, calculating, usually from small population samples, an initial mortality rate (IMR, also known as baseline vulnerability A) and a mortality rate doubling time (MRDT, also known as acceleration of mortality G, [122]). Then, the idea is that “true” aging is reflected by the MRDT, and there is an “aging-independent mortality” as reflected by the IMR. Implicitly, such a distinction sets a threshold at the transition from IMR and MRDT. Moreover, in an approach focusing on health and healthspan, it is the IMR that counts and that we wish to extend, and we may even aim for a high MRDT, to compress the period of morbidity. Consequently, in our framework, there is no such thing as an “aging-independent mortality” [122], consistent with the notion that a biomarker of aging is just a predictor of health and survival that is better than chronological age.

## Conclusion and future work

We here present a framework which defines often used terms in life science research in an integrative manner. We differentiate between states, time periods, underlying biological processes and predictors of the future. We propose to create a framework which enables researchers to apply the terms (and the concepts behind these terms) to different species, for human beings as well as for model organisms used in research on aging and diseases. Taking into account the huge steps basic research has taken in the last years, we also aimed to create the framework as an open and dynamic one which will progress with the growing knowledge on health, aging and disease mechanism and processes. Therefore, the proposed framework should be seen as a starting point because without a precise definition of what we are studying, the results will be less easy to interpret, also from a translational point of view. With this in mind we hope that the proposed framework will help basic researchers and clinicians to gain a deeper understanding of the field and it enables trans- and interdisciplinary research. It will be work of the future to enrich our table of health features by taking into account more operationalizations of health and healthspan from past, present and future studies.

In order to handle the complexity of health features, it would be desirable to develop a formal ontology of these features, in order to enhance interoperability and automated integration of experimental data derived with different subsets of these features. In such a future ontology of health, the features need to be aligned to appropriate top-level classes. It seems to be promising to analyze many physiological functions as processes, considering that the respective dysfunctions consist in the lack of the dispositions to realize these processes [123]. Moreover, our framework is again flexible enough to allow for subtypes of aging, like *linear aging*, or *progressive aging*, in order to single out those aging processes that contribute to a specific kind of decline of health or survival.
